# Molecular Effects of Iodine-Biofortified Lettuce in Human Gastrointestinal Cancer Cells

**DOI:** 10.3390/nu14204287

**Published:** 2022-10-14

**Authors:** Olga Sularz, Aneta Koronowicz, Cayla Boycott, Sylwester Smoleń, Barbara Stefanska

**Affiliations:** 1 Department of Human Nutrition and Dietetics, Faculty of Food Technology, University of Agriculture in Krakow, Balicka 122, 31-149 Krakow, Poland; 2 Food, Nutrition and Health Program, Faculty of Land and Food Systems, The University of British Columbia, 2205 East Mall, Vancouver, BC V6T 1Z4, Canada; 3 Department of Plant Biology and Biotechnology, Faculty of Biotechnology and Horticulture, University of Agriculture in Krakow, Al. 29 Listopada 54, 31-425 Krakow, Poland

**Keywords:** lettuce, iodine, gene expression, DNA methylation, gastrointestinal cancer

## Abstract

Considering the growing number of cancer cases around the world, natural products from the diet that exhibit potential antitumor properties are of interest. Our previous research demonstrated that fortification with iodine compounds is an effective way to improve the antioxidant potential of lettuce. The purpose of the present study was to evaluate the effect of iodine-biofortified lettuce on antitumor properties in human gastrointestinal cancer cell lines, gastric AGS and colon HT-29. Our results showed that extracts from iodine-biofortified lettuce reduce the viability and proliferation of gastric and colon cancer cells. The extracts mediated cell cycle arrest which was accompanied by inactivation of anti-apoptotic Bcl-2 and activation of caspases, as assessed by flow cytometry. However, extracts from lettuce fortified with organic forms of iodine acted more effectively than extracts from control and KIO_3_-enriched plants. Using quantitative PCR, we detected the increase in pro-apoptotic genes *BAD, BAX* and *BID* in AGS cells whereas up-regulation of cell cycle progression inhibitor *CDKN2A* and downregulation of pro-proliferative *MDM2* in HT-29 cells. Interestingly, lettuce extracts led to down-regulation of pro-survival *AKT1* and protooncogenic *MDM2*, which was consistent for extracts of lettuce fortified with organic form of iodine, 5-ISA, in both cell lines. *MDM2* downregulation in HT-29 colon cancer cells was associated with *RB1* upregulation upon 5-ISA-fortified lettuce extracts, which provides a link to the epigenetic regulation of tumor suppressor genes by RB/MDM2 pathway. Indeed, *SEMA3A* tumor suppressor gene was hypomethylated and upregulated in HT-29 cells treated with 5-ISA-fortified lettuce. Control lettuce exerted similar effects on RB/MDM2 pathway and *SEMA3A* epigenetic activation in HT-29 cells. Our findings suggest that lettuce as well as lettuce fortified with organic form of iodine, 5-ISA, may exert epigenetic anti-cancer effects that can be cancer type-specific.

## 1. Introduction

Both colorectal as well as gastric cancer are listed among the most common type of malignancies and rank third and fifth for incidence in the world, respectively [1,2]. Numerous pieces of evidence show that natural plant-derived compounds may contribute to prevention and supporting the treatment of gastrointestinal cancer and other chronic disease [3]. The anticancer potential of the compounds is often related to changes in expression of genes that are implicated in cell cycle arrest and apoptotic death [4]. Among dietary bioactive compounds, polyphenols are best known for their antioxidant, anti-inflammatory and anti-cancer properties, including polyphenols in lettuce [4,5,6,7]. According to existing studies, the trace element iodine also exerts protective effects against tumor progression. It is hypothesized that iodine deficiency may be associated with a heightened risk of gastric, breast and thyroid cancer [8]. Studies indicate that iodine treatment leads to induction of programmed cell death through mitochondrial pathway or generation of iodolipids and also inhibition of cell proliferation [9,10]. Most importantly, iodine has been shown to impact polyphenol content in lettuce which may constitute an indirect mechanism of iodine-mediated anticancer effects [11].

Our previous study indicated a high antioxidant potential of extracts from lettuce fortified with organic forms of iodine [12]. The use of organic iodine, such as 5-iodosalicylic acid (5-ISA), for biofortification of lettuce allows to obtain plants with higher total iodine content as compared to application of inorganic form (KIO_3_) [13]. We also showed that iodine-enriched vegetables have a higher concentration of polyphenols as well as vitamin C and antioxidant enzymes [12]. Moreover, application of these extracts resulted in increasing production of reactive oxygen species (ROS) in gastric and colon cancer cell lines, which may contribute to cells death and anticancer properties [12]. Currently, it is well established that environmental factors lead to accumulation of epigenetic alterations in cells which results in gastrointestinal carcinogenesis [14]. Epigenetic modifications include heritable changes in gene expression that are not caused by altering the DNA sequence. DNA methylation, histone modification, chromatin remodeling complexes and regulation by non-coding RNAs are regarded as known processes of epigenetic mechanisms that are connected to cancer progression [14,15]. It is believed that altered patterns of DNA methylation play a role in the progression of cancer and pathogenesis of other disease [16]. Interestingly, polyphenols from lettuce, iodine, and salicylic acid were all reported to exert certain epigenetic effects [16,17,18,19,20,21,22,23,24,25,26,27].

In this research, we therefore focus on the examination of the molecular changes implicated in anticancer effects of iodine-enriched lettuce extracts in gastrointestinal cancer cells. We performed the analysis of cell cycle and the activity of caspases and Bcl-2 protein in human gastrointestinal cancer cells treated with extracts from iodine-fortified lettuce. We further assessed expression of genes involved in regulation of cell cycle, cell death, survival and oncogenic functions. Our studies indicated the possible epigenetic effects of lettuce extracts leading to activation of methylation-silenced tumor suppressor genes in HT-29 colon cancer cells.

## 2. Materials and Methods

### 2.1. Plant Material and Preparation of Extracts

The research material was lettuce *L. sativa* cv. ‘Melodion’ that was cultivated and fortified with inorganic, KIO_3_, and organic, 5-ISA and 3,5-diISA, forms of iodine, as described in our previous research [12,13]. Non-enriched lettuce was used as control lettuce. Ethanolic extracts from iodine-biofortified lettuce were prepared by extraction of the plant leaves with 70% ethanol, rotary evaporation and lyophilization. The freeze-dried extracts from lettuce were stored at −20 °C for further analysis.

### 2.2. Cell Culture

This study was carried out with use of three human cell lines: normal colon epithelial cell line CCD 841 CoN (ATCC^®^ CRL-1790™, Manassas, VA, USA), gastric adenocarcinoma cell line AGS (ATCC^®^ CRL-1739™, Manassas, VA, USA) and human colorectal adenocarcinoma cell line HT-29 (ATCC^®^ HTB-38™, Manassas, VA, USA). These cell lines were obtained from the American Type Culture Collections (ATCC, Manassas, VA, USA). Cell cultures were maintained under controlled conditions (temperature 37 °C; atmosphere: air 95%; CO_2_ 5%;) in appropriate medium supplemented with 10% FBS according to the ATCC protocol.

### 2.3. Cell Treatment

To assess the concentration of extracts from iodine-biofortified lettuce that leads to 10% cell growth inhibition (IC_10_) as compared with cells treated with non-biofortified lettuce (control lettuce), cytotoxicity, cell viability and proliferation assays were performed. CCD 841 CoN, AGS and HT-29 cells were seeded at a density of 8 × 10^4^ cells/well in a 96-well microtiter plates and incubated in growth medium for 24 h. After this time, the growth medium was renewed and substituted with a medium containing extract from control lettuce and iodine-biofortified lettuce at the concentration of 1000 µg/mL for 24, 48 and 72 h. Non-treated cells were used as a negative control.

For cell cycle, Bcl-2 activity and multicaspase activity assays, 24 h prior to treatments, cancer cells were plated at a density of 3 × 10^5^ cells per well in 6-well plates. Cell were then treated for 24 h with synthetic iodosalicylic acids or extracts from control lettuce and lettuce biofortified with organic (5-ISA and 3,5-diISA lettuce) and inorganic (KIO_3_ lettuce) forms of iodine. To analyze gene expression, cancer cells were seeded into cell culture dishes (6 × 10^5^ cells/dish) overnight and treated with extracts from control, 5-ISA and 3,5-diISA lettuce (1000 µg/mL) for 48 h. Cells grown in only complete growth medium were used as a negative control, whereas cells treated with a pro-apoptotic compound, staurosporine (1.5 µM for 3 h), served as a positive control.

### 2.4. Cell Cytotoxicity

Cell cytotoxicity was analyzed for extracts from control and iodine-biofortified lettuce at one point of time (24 h) by the Cytotoxicity Detection Kit (Lactate dehydrogenase, LDH) (Roche, BASEL, Switzerland), according to the instructions given by the manufacturer.

Cell viability was assessed with the crystal violet assay, that is based on staining adherent cells that are attached to microtiter plate. After 24, 48 and 72 h incubation with extracts from control and iodine-biofortified lettuce, cells were washed with 1 × phosphate-buffered saline (PBS), fixed with methanol for 15 min and stained with 0.5% crystal violet. Then, the plates were washed three times with distilled water and dried in the air. The absorbance of the sample was measured after 30 min of incubation with elution solution (0.1 M citrate buffer dissolved in methanol and H_2_O) at 540 nm using a plate reader Thermo Scientific™ Multiskan™ GO Microplate Spectrophotometer (Waltham, MA, USA). Results were standardized to the negative control (non-treated cells) as 100%.

### 2.5. The Impact on Cell Growth

Cell proliferation was determined by using commercial colorimetric and immunoassay 5-Bromo-2’-deoxy-uridine Labeling and Detection Kit III (Roche, Basel, Switzerland), that is based on the measurement of BrdU incorporation during DNA synthesis. The assay was carried out according to the manufacturer’s protocol. All results were standardized to the negative control (non-treated cells) as 100%.

### 2.6. Cell Cycle Analysis

Effect of extracts from control and iodine-biofortified lettuce on cell cycle in cancer cells was determined by using Muse™ Cell Cycle Kit (Merck Millipore, Billerica, MA, USA, Catalog No. MCH100106). The kit is based on the nuclear DNA intercalating stain-propidium iodide (PI) and allows quantitative measurements of the percentage of cells in the three phases of cell cycle: G0/G1, S, and G2/M. The results of the cell cycle analysis were measured using the Muse™ Cell Analyzer (Merck Millipore, USA).

### 2.7. Bcl-2 Activity Analysis

The level of cells expressing Bcl-2 was measured with the Muse™ Bcl-2 Activation Dual Detection Kit (Merck Millipore, USA, Catalog No. MCH200105) by using pair of antibodies that enables detection of total protein and phosphorylated form. This assay provides information about percentage of: inactivated cells (expressing Bcl-2 but non-phosphorylated), activated cells (phosphorylated Bcl-2) and non-expressing cells. Analysis was performed using the Muse™ Cell Analyzer (Merck Millipore, USA).

### 2.8. Multicaspase Activity Analysis

The Muse™ MultiCaspase Kit (Merck Millipore, Billerica, MA, USA, Catalog No. MCH100109) was used to detect the activity of multiple caspases (caspase-1, 3, 4, 5, 6, 7, 8, 9) in cancer cells. Due to using a derivatized VAD-peptide and a dead cell dye, the assay allows for identification of the activity of caspases and distinguishes four population of cells: Live, Caspase (+), Caspase+/Dead and Dead. The results of the assay were measured using the Muse™ Cell Analyzer (Merck Millipore, Billerica, MA, USA).

### 2.9. RNA Isolation, RT and Real-Time PCR Analysis

The total RNA was isolated from cancer cell lines using the Total RNA Mini Kit (A&A Biotechnology, Gdansk, Poland), in accordance with the manufacturer’s instruction. Concentration, purity and quality of isolated RNA was measured in µDrop Plate (Thermo Fisher Scientific, Waltham, MA, USA). To synthesize cDNA, the reverse transcriptase (RT) reaction was performed using iScript Reverse Transcription Supermix (Bio-Rad, Hercules, CA, USA). A quantitative analysis of gene expression was carried out in CFX96 Touch^™^ Real-Time PCR Detection System instrument on prime PCR custom plates with the use of SsoAdvanced™ Universal SYBR^®^ Green Supermix (Bio-Rad, Hercules, CA, USA). Amplification was performed using the following conditions: activation at 95 °C for 2 min, 40 cycles of denaturation at 95 °C for 5 sec, annealing at 60 °C for 30 sec and melt curve at 65–95 °C for 5 sec. Two reference genes (GAPDH and ACTB) were used for normalization and the relative expression level were calculated with use the ΔΔCT method. Amplicon Context Sequence for all analyzed genes and primer sequences for SEMA3A are listed in Supplementary Table S1 and Table S2, respectively.

### 2.10. DNA Isolation and Pyrosequencing

DNA was isolated from untreated cells (negative control) and cell treated with 1000 µg/mL of the extracts from control lettuce and lettuce biofortified with 5-ISA using a standard phenol:chloroform extraction method. Bisulfite conversion of DNA was performed as previously described [28]. Briefly, 2 mg of DNA was digested with EcoRI for 3 h at 37 °C, followed by purification using the Quick Clean PCR Purification Kit (GenScript, Piscataway, NJ, USA) and denaturation with 3 M NaOH for 15 min at 37 °C. Freshly prepared 3.6 M sodium bisulfite/1 mM hydroquinone mixture (pH 5.0) was added to denatured DNA and the samples were incubated for 2 min at 95 °C, 8 h at 55 °C, and finally 2 min at 95 °C and 2 h at 55 °C, to complete bisulfite conversion. Following second denaturation and purification (Quick Clean PCR Purification Kit, GenScript, Piscataway, NJ, USA), DNA was precipitated with 95% ethanol and resuspended in *TE* buffer.

Bisulfite converted DNA was amplified using HotStar Taq DNA polymerase (Qiagen, GmbH, Hilden, Germany) and biotinylated primers specific to a target *SEMA3A* region (please see Supplementary Table S3 for primer sequences). Biotinylated DNA strands were processed in the PyroMark Q48 Autoprep instrument (Qiagen, GmbH, Hilden, Germany) which uses sequencing by synthesis principle. The percentage of DNA methylation at a single CpG site resolution was calculated by processing the sequencing data in PyroMark Q48 software (Qiagen, GmbH, Hilden, Germany).

### 2.11. Statistical Analysis

The statistical analysis was performed by using Statistica 13.1 PL program (StatSoft, Inc., Tulsa, OK, USA). All experiments were performed in at least in three technical and biological replications. The Shapiro–Wilk test for normality was conducted to evaluate whether the data follow a normal distribution. Results are expressed as mean ± standard deviation (SD). Statistically significant differences were assessed using analysis of variance (ANOVA) followed by Tukey’s post hoc and t-test. p values less than 0.05 were considered as statistically significant.

## 3. Results

### 3.1. Cell Cytotoxicity

Cytotoxic effects of extracts from control and iodine-biofortified lettuce in human gastrointestinal cell lines (AGS, HT-29) and normal colon epithelial cell line (CCD 841 CoN) were evaluated upon 24 h of incubation (Table 1). Treatment with lettuce extracts at the concentration of 1000 µg/mL did not cause a cytotoxic effect and necrosis in cancer and normal cells. The lowest cytotoxicity level was observed in HT-29 cells. However, the number of killed cells was below 10% in all tested cell lines. Therefore, the concentration of 1000 µg/mL was selected to carry out further analyses in the three cell lines.

**Figure 1:**
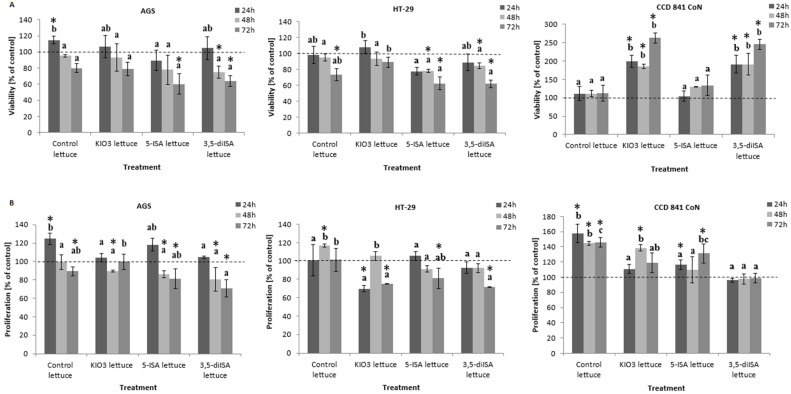
The effect of extracts from iodine-biofortified lettuce on cell viability (**A**) and proliferation (**B**) in human gastrointestinal cell line AGS, colorectal adenocarcinoma cell line HT-29 and normal colon epithelial cell line CCD 841 CoN. Cells were treated for 24, 48 and 72 h with 1000 µg/mL extracts from control lettuce, or KIO3- fortified lettuce, or 5-ISA-fortified lettuce, or 3,5-diISA-fortified lettuce. Statistical significance was assessed using one-way ANOVA followed by Tukey’s post hoc. Columns with the same letter are not significantly different (*p* > 0.05). * differences statistically significant relative to the negative control sample (NC) when *p* < 0.05 (*t*-test).

Extracts from iodine-fortified lettuce inhibited cancer cell viability and proliferation at a concentration of 1000 µg/mL in a time-dependent manner (Figure 1). In AGS cell lines, 72 h-treatment with extracts from 5-ISA and 3,5-diISA-fortified lettuce showed a statistically significant decrease in cell viability by approximately 40%, as compared to untreated cells (negative control) (Figure 1A). Interestingly, the reduction of cell viability was not observed in AGS cells after incubation with control- and KIO_3_-fortified lettuce extracts. Similar results were observed in HT-29 cell line, where 72 h-exposure to lettuce with iodosalicylic acids resulted in a significant 40% reduction of cell viability (Figure 1A).

### 3.2. The Impact on Cell Growth

The results obtained from the BrdU Cell Proliferation Assay revealed that the incubation with extracts from iodine-enriched lettuce significantly reduces cell growth of HT-29 cells after 48 and 72 h (Figure 1B). The highest 30% decrease in proliferation was evident after 72 h of incubation with 3,5-diISA-fortified lettuce in both AGS and HT-29 cells. In AGS cells, the application of extracts from lettuce enriched with inorganic form of iodine did not reduce cell proliferation, as compared with control lettuce. However, HT-29 cells treated with KIO_3_-fortified lettuce exhibited reduced proliferation by approximately 25%. In human normal colon epithelial CCD 841 CoN cells, the inhibitory effect of treatments with iodine-fortified lettuce was not observed (Figure 1B).

Prior to carrying out further studies (caspases, Bcl-2, cell cycle analysis and gene expression), we performed Muse™ Annexin V & Dead Cell Assay (Merck Millipore, Billerica, MA, USA, Catalog No. MCH100105) to determine the cell death mechanism (Supplementary Figure S4). We observed that incubation for 48 to 72 h increased the necrotic (dead) cell population to about 50%. Apoptotic death suppresses cancer progression, while necrotic death through secretion of necrotic factors may potentially promote cancer development. Our research concerns the natural plant products that may be helpful in preventing and supporting the treatment, however they are not anti-cancer drugs. Therefore, to induce cancer cell death via apoptosis we decided to use a shorter time of incubation for further analysis.

### 3.3. Lettuce Extracts Reduce Cell Cycle Progression

**Figure 2:**
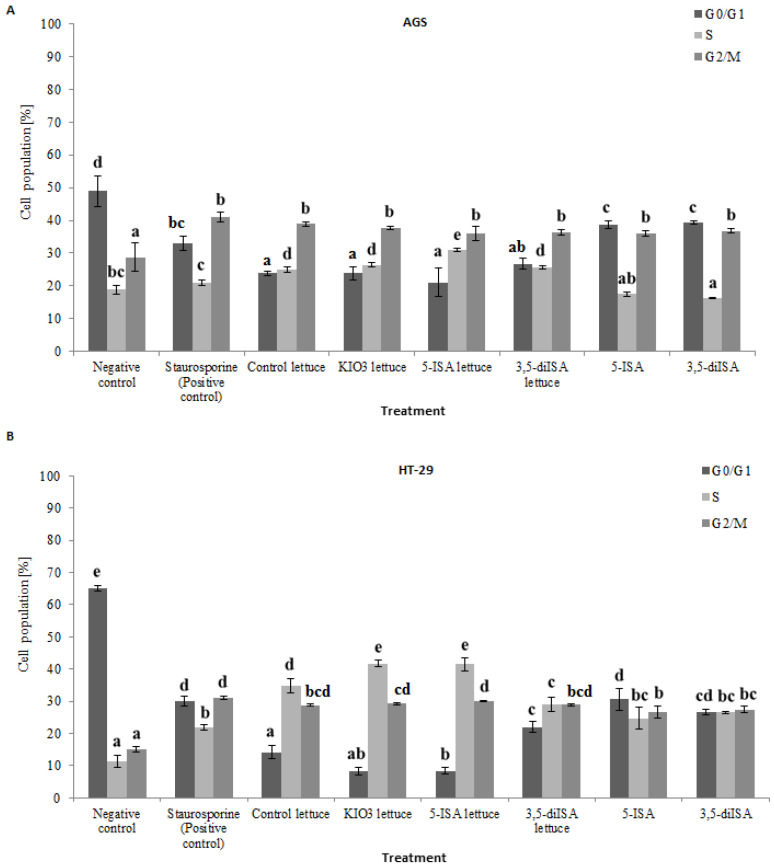
The effect of extracts from iodine-biofortified lettuce on cell cycle in human gastrointestinal cell line AGS (**A**) and colorectal adenocarcinoma cell line HT-29; (**B**). Cells were treated for 24 h with 1000 µg/mL extracts from control lettuce, or KIO3- fortified lettuce, or 5-ISA-fortified lettuce, or 3,5-diISA-fortified lettuce, or synthetic 5-ISA, or synthetic 3,5-diISA, or 1.5 µM staurosporine as positive control. Statistical significance was assessed using one-way ANOVA followed by Tukey’s post hoc. Columns with the same letter are not significantly different (*p* > 0.05).

To examine whether the reduction in viability and inhibition of cell proliferation of AGS and HT-29 cells is associated with cell cycle arrest, a cell cycle analysis was performed (Figure 2 and Supplementary Figure S1). Figure 2 depicts the distribution of gastric and colon cancer cells within each phase of cell cycle, G0/G1, S, and G2/M, after iodine-biofortified lettuce treatments, as quantified based on flow cytometry results in Supplementary Figure S1. In both tested cancer cell lines, extracts from iodine-fortified lettuce led to significant changes in cell cycle distribution, as compared to untreated cells (negative control). Treatment of HT-29 cells with 1000 µg/mL extracts from 5-ISA and 3,5-diISA fortified lettuce induced S phase cell cycle arrest (Figure 2B). The percentage of HT-29 cell population in S phase was only 10% in negative control, and significantly increased to almost 42% upon KIO_3_- and 5-ISA-biofortified lettuce and to nearly 29% after incubation with 3,5-diISA-biofortified lettuce extracts (Figure 2B). In AGS cells, 24 hr-exposure to 5-ISA- and 3,5-diISA-enriched lettuce resulted in 25–31% of cells in S phase, as compared to 20% in negative control (Figure 2A). The largest population of AGS cells treated with 5-ISA- and 3,5-diISA-fortified lettuce was detected in G2/M phase (Figure 2A). Flow cytometry also revealed that the incubation of AGS cells with synthetic 5-ISA or 3,5-diISA leads to decrease in the number of cells in S phase as compared to control lettuce. The opposite effect was observed in HT-29 cells where S phase population significantly increased upon treatments with synthetic 5-ISA or 3,5-diISA, as compared to negative control (Figure 2).

### 3.4. Activity of Anti-Apoptotic Bcl-2 Is Reduced upon Lettuce Extracts

**Figure 3:**
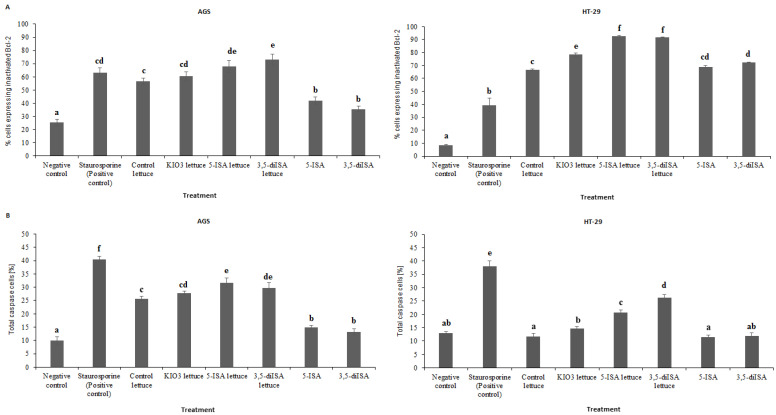
The effect of extracts from iodine-biofortified lettuce on Bcl-2 expression: inactivated cells (**A**) in human gastrointestinal cell line AGS and colorectal adenocarcinoma cell line HT-29. (**B**) The effect of extracts from iodine-biofortified lettuce on activity of caspases in human gastrointestinal cell line AGS and colorectal adenocarcinoma cell line HT-29. Cells were treated for 24 h with 1000 µg/mL extracts from control lettuce, or KIO3- fortified lettuce, or 5-ISA-fortified lettuce, or 3,5-diISA-fortified lettuce, or synthetic 5-ISA, or synthetic 3,5-diISA, or 1.5 µM staurosporine as positive control. Statistical significance was assessed using one-way ANOVA followed by Tukey’s post hoc. Columns with the same letter are not significantly different (*p* > 0.05).

To assess the impact of extracts from iodine-biofortified lettuce on the levels of anti-apoptotic Bcl-2 protein in human gastrointestinal cancer cell lines, the Muse™ Bcl-2 Activation Dual Detection Assay (Merck Millipore, Billerica, MA, USA) was used (representative plots in Supplementary Figure S2). In AGS gastric cancer cells (AGS) as well as HT-29 colon cancer cells, we detected a statistically significant increase in cells expressing inactivated Bcl-2 after treatment with iodine-biofortified lettuce extracts, as compared to the control lettuce or negative control (Figure 3A). In AGS cells, 68% and 73% of cells expressed inactivated Bcl-2 protein upon 24 h-treatment with 5-ISA- and 3,5-diISA-fortified lettuce, respectively. These results represent a statistically significant increase from 58% of cell population in control lettuce and 28% in negative control (Figure 3A). In HT-29 cells, the level of cell population expressing inactivated Bcl-2 rose from 18% in negative control and 64% in control lettuce to approximately 92% after 24 h-incubation with 5-ISA and 3,5-diISA-fortified lettuce extracts. The treatment with synthetic 3,5-diISA also led to a significantly higher percentage of cells expressing inactivated Bcl-2 in HT-29 cell line, as compared with control lettuce and negative control (Figure 3A). Similar increases were observed for extracts of lettuce fortified with inorganic iodine, KIO_3_, however the change was statistically insignificant in AGS cells when compared with control lettuce. Interestingly, the levels of cells with inactivated Bcl-2 were higher in colon HT-29 cancer cells than in AGS gastric cancer cells across different conditions.

### 3.5. Pro-Apoptotic Caspases Are Activated by Lettuce Extracts

Following the assessment of Bcl-2 levels, we proceeded with evaluating the profile of the activity of other proteins involved in cell death, namely caspases 1, 3, 4, 5, 6, 7, 8 and 9, after treatment with fortified lettuce extracts, using the Muse^TM^ MultiCaspase Assay (Merck Millipore, Billerica, MA, USA) (representative plots in Supplementary Figure S3). In AGS and HT-29 cell lines, exposure to 1000 µg/mL extracts from lettuce biofortified with inorganic and organic forms of iodine, resulted in the increase in total caspase activity, as compared with negative control and control lettuce, after 24 h of incubation (Figure 3B). In AGS cells, the percentage of cells with active caspases rose from 10% in negative control and 25% in control lettuce to almost 32% after treatment with 5-ISA and 3,5-diISA-fortified lettuce and to 28% after KIO_3_-fortified lettuce. In HT-29 cells, we detected 2.1- and 2.7-fold increase in the number of caspase-positive cells upon treatment with 5-ISA and 3,5-diISA-fortified lettuce, respectively, relative to negative control or control lettuce (Figure 3B). Synthetic forms of iodine, 5-ISA and 3,5-diISA, induced caspases to a higher extent in AGS cells than in HT-29 cells, as depicted by a higher percentage of caspase-positive cells (Figure 3B).

### 3.6. Lettuce Extracts Impact Expression of Genes Involved in Cell Proliferation, Apoptosis and Oncogenic Signaling Pathways 

**Figure 4:**
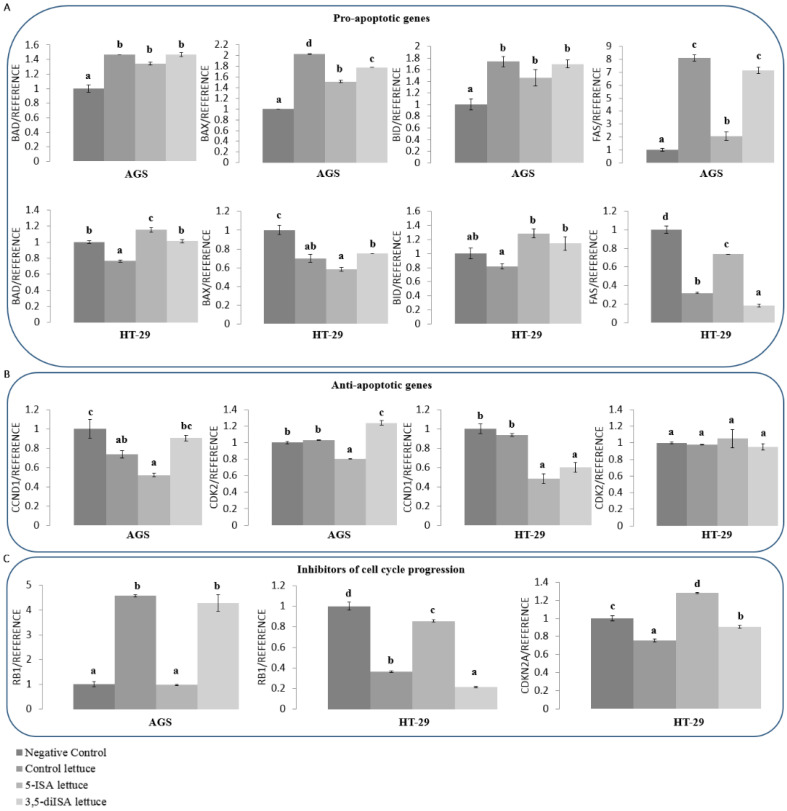
The effect of extracts from iodine-biofortified lettuce on mRNA expression of genes in human cancer cells of AGS and HT-29 cell lines. (**A**) Pro-apoptotic genes. (**B**) Anti-apoptotic genes (**C)** Inhibitors of cell cycle progression. Cells were treated for 48 h with 1000 µg/mL extract from control lettuce, or 5-ISA-fortified lettuce, or 3,5-diISA-fortified lettuce. The results are shown as means ± SD. Statistical significance of treatment: Statistical significance was assessed using one-way ANOVA followed by Tukey’s post hoc. Columns with the same letter are not significantly different (*p* > 0.05).

Changes in cell cycle distribution and the activity of apoptosis-related proteins upon treatment with iodine-fortified lettuce suggested possible effects on expression of genes implicated in regulation of these biological processes. Therefore, we assessed mRNA levels of a set of genes as shown in Figure 4, using qRT-PCR. Since detected effects on the inhibition of cell viability, inhibition of cell cycle progression and induction of apoptosis-related proteins were more profound upon organic iodine-fortified lettuce, we proceeded with mRNA measurements in cells treated with 5-ISA- and 3,5-diISA-fortified lettuce.

In AGS gastric cancer cells, we observed consistent 1.3-8-fold up-regulation of pro-apoptotic genes such as *BAD, BAX, BID*, and *FAS* after control lettuce and lettuce fortified with organic iodine, as compared to negative control (Figure 4A). Simultaneously, anti-apoptotic *CCND1* and *CDK2* were downregulated by 48% and 20% upon 5-ISA-fortified lettuce, respectively. In HT-29 colon cancer cells, only the extract from 5-ISA-fortified lettuce led to significant 1.2-fold upregulation of pro-apoptotic *BAD* while anti-apoptotic *CCND1* was downregulated by approximately 52% and 40% upon both 5-ISA- and 3,5-diISA-fortified lettuce, respectively, as compared to negative control (Figure 4A,B). Of note, treatment of HT-29 cells with control lettuce resulted in downregulation of *BAD, BAX* and *FAS* which was partially rescued by iodine-fortified lettuce. These results indicate apparent differences in apoptotic pathways between the cell lines of distinct cancer type.

Tumor suppressor gene, *CDKN2A*, was detected only in HT-29 cells and was upregulated by 28% vs. negative control and by 53% vs. control lettuce upon treatment with 5-ISA-fortified extracts (Figure 4C). *CDKN2A* blocks cell cycle progression similarly to *RB*, which was increased by the same extract but only vs. control lettuce in HT-29 cells. In contrast, control lettuce and lettuce fortified with 3,5-diISA profoundly increased *RB* mRNA levels in AGS cells (Figure 4C).

**Figure 5:**
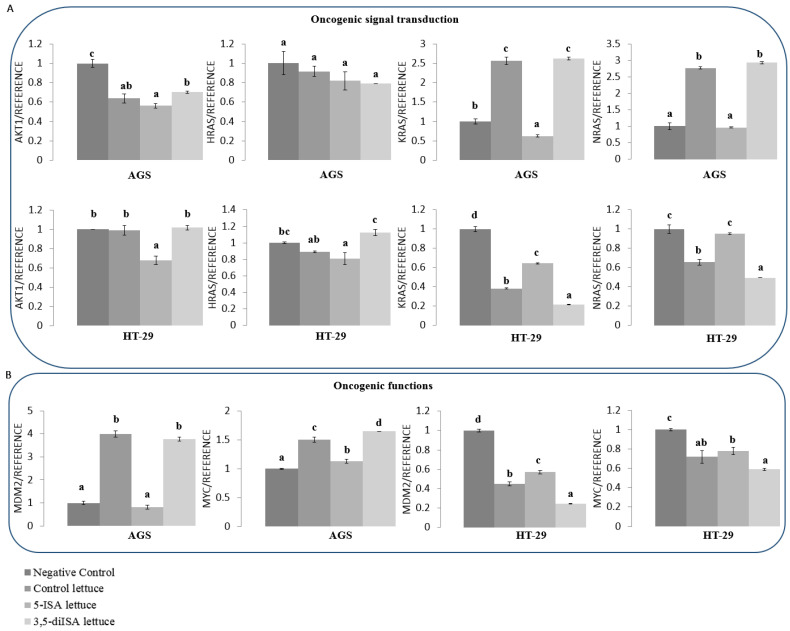
The effect of extracts from iodine-biofortified lettuce on mRNA expression of genes in human cancer cells of AGS and HT-29 cell lines. (**A**) Oncogenic signal transduction. (**B**) Oncogenic functions. Cells were treated for 48 h with 1000 µg/mL extract from control lettuce, or 5-ISA-fortified lettuce, or 3,5-diISA-fortified lettuce. The results are shown as means ± SD. Statistical significance was assessed using one-way ANOVA followed by Tukey’s post hoc. Columns with the same letter are not significantly different (*p* > 0.05).

Treatment with control lettuce and iodine-fortified lettuce led to downregulation of several key oncogenes, including those involved in oncogenic signaling pathways (Figure 5). *AKT1*, which drives activation of oncogenic PI3K pathway, was downregulated by approximately 30–43% upon each of the extracts in AGS cells but only by 5-ISA-fortified lettuce in HT-29 cells, as compared with negative control (Figure 5A). *KRAS* and *NRAS* oncogenes from MAPK signaling pathway were downregulated by 5-ISA-fortified lettuce in AGS cells, as compared with negative control. More consistent mRNA reduction of oncogenes, including *KRAS* (by 40–80% vs. negative control), *MDM2* (by 40–80% vs. negative control), and oncogenic transcription factor *Myc* (by 20–40% vs. negative control) was detected upon each of the extracts in HT-29 cells (Figure 5A,B). Strikingly, *MDM2* was 1.5-fold upregulated by control lettuce in AGS cells and treatment with 5-ISA-fortified lettuce brought *MDM2* levels down to levels in untreated cells (negative control) (Figure 5B). Of interest, *MDM2* is implicated in promotion of cell cycle progression and inhibition of apoptosis, as well as in the epigenetic silencing of tumor suppressor genes through RB/DNMT3A-dependent pathway [29].

### 3.7. Extract from 5-ISA-Fortified Lettuce Leads to Hypomethylation and Up-Regulation of SEMA3A Tumor Suppressor Gene

**Figure 6:**
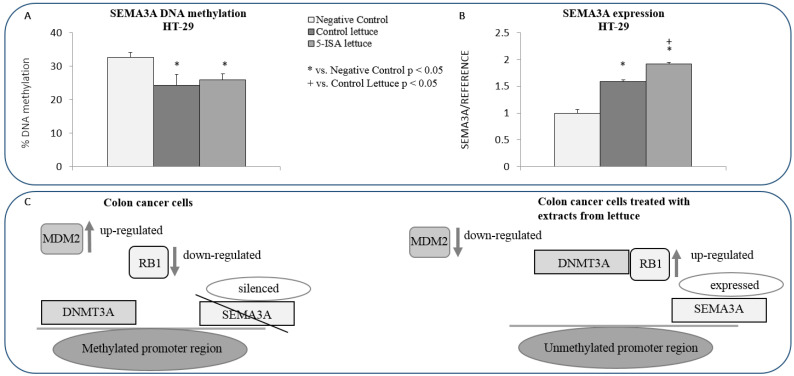
The effect of extracts from iodine-biofortified lettuce on DNA methylation (**A**) and expression of *SEMA3A.* (**B**) in human cancer cells of HT-29 cell line. (**C**) Schematic of proposed mechanism of hypomethylation and upregulation of *SEMA3A* tumor suppressor gene by extract from 5-ISA-fortified lettuce. Cells were treated with 1000 µg/mL extract from control lettuce, or 5-ISA-fortified lettuce. The results are shown as means ± SD. Statistical significance of treatment was assessed using t-test * vs. negative control when *p* < 0.05; + vs. control lettuce when *p* < 0.05.

*MDM2* has been shown to contribute to silencing of tumor suppressor genes through *DNMT3A*-dependent promoter methylation. Once *MDM2* is overexpressed, *RB* expression drops and *RB*-mediated suppression of *DNMT3A* activity is diminished, which results in overactive *DNMT3A* catalyzing DNA methylation and silencing of tumor suppressor genes [29]. In our present study, *MDM2* downregulation in HT-29 colon cancer cells (Figure 5B) was associated with *RB1* upregulation upon 5-ISA-fortified lettuce extracts as compared to control lettuce (Figure 4C). We therefore proceeded with investigating whether there is a link between the effects of 5-ISA-fortified extract and the epigenetic regulation of tumor suppressor genes by RB/MDM2 pathway. We focus the study on a tumor suppressor gene, *SEMA3A*, as *SEMA3A* promoter was shown to be bound and methylated by *DNMT3A* in our previous study [30]. We detected 10% decrease in DNA methylation at *SEMA3A* promoter in HT-29 cells treated with 5-ISA-fortified lettuce (Figure 6A), which coincided with nearly 2-fold upregulation of *SEMA3A*, as compared with negative control (Figure 6B). Although control lettuce and 5-ISA-fortified lettuce led to *SEMA3A* hypomethylation to a similar extent, *SEMA3A* expression was significantly higher upon 5-ISA-fortified lettuce than after control lettuce.

## 4. Discussion

Iodine has been shown to possess antiproliferative activities in different types of cancer, including colon, breast, lung, and pancreas [31,32]. However, existing studies mainly focus on anticancer properties of nascent iodine, whereas investigations with food containing inorganic/organic forms of iodine are limited [31]. According to the World Health Organization, nutritional supplementation strategy through production of biofortified food is an important and effective way allowing the reduction of micronutrient malnutrition. Currently, there are many food vehicles that are commonly fortified with iodine, including water, sugar, cereal grains, and vegetable oils [33]. It was also shown, that iodine fortification of plants such as lettuce, tomato, potato, and carrots can significantly improve iodine intake because leafy vegetables can be consumed without cooking which consequently reduces the loss of iodine [34]. Our previous research confirmed that fortification of lettuce with organic forms of this micronutrient, i.e., iodosalicylates 5-ISA and 3,5-diISA, can be an effective way to increase iodine concentration in crop plants. We also demonstrated that the elevation of iodine level in fortified plants may contribute to production of ROS and presumably enhanced anticancer activity of fortified plants [13]. In the present work on gastrointestinal cancer cells, we examine the molecular impact of extracts from lettuce that was fortified with inorganic iodine and organic forms of iodosalicylates.

Iodosalicylates (5-ISA and 3,5-diISA) can be naturally synthesized by plants. These compounds are also present in soil environment and roots can take exogenous 5-ISA and 3,5-diISA from soil solution. Moreover, lettuce roots can take up exogenous iodosalicylates from the nutrient solution in hydroponic cultivation, which is the case in our present study. In our model, lettuce may contain both endogenous and exogenous 5-ISA and 3,5-diISA (especially in plants enriched with these compounds). Both 5-ISA and 3,5-diISA in lettuce plants are involved in the metabolism of other organic iodine compounds in this plant, such as plant-derived thyroid hormone analogs (PDTHA), i.e., triiodothyronine (T3) and thyroxine (T4). In a study by Smoleń et al. [35], a theoretical metabolic pathway of iodosalicylates has been described, along with the interpretation of the biochemical response of human cells to iodine-enriched plant extracts containing many different organic iodine compounds. Iodosalicylates can operate in a cyclic system as well as other cycles of cellular processes. In addition, chemical reactions where iodine reacts with itself are described in the literature as well. Therefore, it is difficult to establish whether 5-ISA and 3,5-diISA are likely to originate iodide or iodine

Koronowicz et al. [36] in their previous study, explained that the action of iodine is mediated by thyroglobulin (TG), whose tyrosine residues are iodinated in the synthesis pathway of thyroid hormone (TH). Their study demonstrated that covalently bound iodine forms are present in biofortified lettuce that may have effects on TG produced in thyroid glands. In mammalian cells, iodine uptake depends on the level of expression of the sodium iodide symporter (NIS) [37]. In vivo studies have shown that breast cancer cells have increased NIS expression, iodine uptake and ability to transport iodine in human breast tumors [38]. Therefore, in hormone-dependent cell lines, iodine can inhibit cancer progression through modulation of the estrogen pathway and changes in expression of genes involved in hormone metabolism and proliferation [39]. NIS expression was also demonstrated in small intestines and gastric mucosa, therefore applied iodine-fortified lettuce can effectively reduce the viability and proliferation of gastric (AGS) and colon (HT-29) cancer cells.

In this study, we applied different assays for the assessment of cytotoxicity of the extracts. In Table 1, we showed results from LDH assay that measures cytosolic lactate dehydrogenase enzyme (LDH). LDH is released from damaged cells due to plasma membrane disruption which is characteristic of necrosis rather than apoptosis. Therefore, LDH assay allows to enumerate the percentage of dead cells and a necrotic potential of the extracts. In another assay we used, the Crystal Violet assay, viable cells that are attached to cell culture dishes are stained with the dye while dead detached cells are washed away. Hence, viability is measured rather than cell death and cell death is estimated based on a comparison with experimental controls. The discrepancies between the results obtained in these two assays may result from their distinct principles in measuring cell viability/death. Elengoe and Hamdan [40] demonstrated that the most sensitive cell viability tests were those measuring the cells attached to the plate surface (crystal violet). A similar conclusion was made in Mickuviene et al. [41], where it was shown that the sensitivity of the crystal violet assay was higher than LDH and MTT assays, which is consistent with our results.

Our findings showed that extracts from iodine-fortified lettuce decrease viability and proliferation of human AGS and HT-29 cancer cell lines. However, the effects were time- and cell-dependent and varied between iodine forms (Figure 1). In AGS cells, only extracts from organic iodine-fortified lettuce decreased cell viability, as compared with negative control, and this effect was observed only upon 72 h-incubation (Figure 1A). Extracts were more potent in HT-29 cells, where reduced cell viability was detected after 72 h-treatment with each studied extract, except lettuce fortified with inorganic iodine that produced no effect (Figure 1A). Only extracts from organic iodine-fortified lettuce decreased the number of viable cells upon 48 h-exposure. The impact on cell proliferation was more mixed. However, extracts with 3,5-diISA-lettuce consistently led to reduced proliferation in HT-29 cancer cell line relative to negative control and control lettuce (Figure 1B). Results obtained by Koronowicz et al. [36] also showed time-dependent inhibition of colon cancer cell proliferation after treatment with KI-fortified lettuce extract with no effects upon treatment with KI only. Interesting effects were observed by Rösner et al. [31], who found a significant dose-dependent antiproliferative effect of povidone-iodine (PVP-I) in melanoma IPC, breast carcinoma MCF-7, lung A549 and bronchial H1299 cancer cell lines after 6 days of treatment. PVP-I, commonly used as an antiseptic agent, is a complex of iodine and povidone, in which povidone component acts as a carrier of free iodine and deliver it to target cells [42]. These results confirm that organic as well as inorganic forms of iodine (PVP-I and a combination of iodine and the ionic state of iodine, namely iodide) act as an inhibitor of cancer cell proliferation [31]. Antiproliferative and apoptotic effects may be explained by the fact that in cancer cells molecular iodine (I_2_) binds membrane lipids and generates iodolipids which are involved in activation of apoptotic pathways and inhibition of genes related to survival and chemoresistance [43]. Furthermore, it was found that iodolipids reduce the expression of markers associated with the tumor formation and metastasis [44,45]. In vitro studies confirmed that molecular iodine treatment decreases the proliferation rate and increases apoptosis in cancer cells [45]. Anticancer effect of iodine is observed only in those organs that are able to uptake it and the efficacy depends on the ability of cells to take up different forms of iodine. It is known that beside thyroid, also gastric, colon and breast tissues has capacity to uptake I^-^ and tumorigenesis may reduce or eliminate this process. There are in vitro and in vivo studies, according to which molecular iodine (I_2_), rather than iodide (I^-^), is responsible for protective effects against cancer [46]. Research in thyroid and breast cancer showed that to induce apoptosis process, it is necessary to enzymatically oxidized I^-^ to I_2_. Gastrointestinal cells similarly like prostate epithelial cells exhibit peroxidase activity which allow for the transformation from iodide to iodine [9,47]. Aranda et al. [9] indicated that cancer cells have the ability to reactivate proliferation after discontinuation of iodine treatment, which confirms its protective effect against cancer progression. Iodine may influence apoptosis presumably directly through oxidant/antioxidant activity (mitochondrial pathway) or indirectly through iodolipid formation and activation of PPAR-γ (peroxisome proliferator-activated receptor) [9,46].

Our research clearly demonstrates more profound inhibition of HT-29 cancer cells growth upon treatment with extracts from lettuce fortified with 5-ISA and 3,5-diISA, as compared with inorganic form and/or lettuce alone (Figure 1). We suspect that more potent activities of extracts with organic iodine might be linked to salicylic acid that is known to have therapeutic properties. Dachineni et al. [48] for the first time showed that salicylic acid metabolites and derivatives exert pro-apoptotic effects and inhibit the activity of cyclin-dependent kinases (CDKs) interfering in cell cycle progression of colorectal carcinoma cells. CKDs are key cell cycle regulatory proteins which through binding to cyclins, enable the progression to the next phase of cell-division cycle.

In this study, we assessed the effect of iodine treatment on the cell cycle distribution of AGS and HT-29 cancer cells. Extracts from control lettuce and iodine-biofortified lettuce led to increased S phase population in AGS and HT-29 cells with simultaneous decrease in G0/G1 population, as compared with negative control (Figure 2). This indicates that lettuce with and without iodine brings about cell cycle arrest after 24h of treatment. However, no differences were detected in cell proliferation at this incubation time. To determinate proliferation we used Cell Proliferation ELISA BrdU Assay, that is based on the measurement of thymidine analogue 5-bromo-2′-deoxyuridine (BrdU) incorporation into DNA. BrdU is incorporated into DNA during DNA synthesis and therefore during the S phase of a cell cycle. In AGS cells, the largest population of cells treated with 5-ISA- and 3,5-diISA-fortified lettuce was in G2/M phase. In HT-29 cells, we observed statistically significant increase in the percentage of S and G2/M phase cells. Therefore, we could not detect differences in BrdU assay, because the arrest of cells occurs in this or next phase of cell cycle.

In contrary, the study by Zhou et al. [49] demonstrated that lettuce extracts led to an increase in the percentage of Caco-2 cells in G1 phase. This discrepancy could be associated with a basal distribution of Caco-2 cells across the phases of the cell cycle, a dose and time of treatments as well as with differences in phenolic constituents of the extracts. Indeed, they showed that phenolic compounds in lettuce extracts have distinct effects on cell cycle arrest [49]. In our study, we also observed accumulation of cells in G2/M phase upon lettuce extracts, suggesting induction of cell death. Interestingly, the effects were more profound in HT-29 colon cancer cells, where also treatments with lettuce fortified with inorganic iodine and organic 5-ISA form resulted in higher number of cells in S phase as compared with control lettuce (Figure 2B). 

Our previous research showed that treatment with iodine-fortified lettuce extracts increases levels of intracellular reactive oxygen species (ROS) in cancer cells HT-29 and AGS [12]. However, we also provided the evidence that iodine-enriched lettuce extracts have no impact on increasing the ROS level in normal colon epithelial cell line (CCD 841 CoN). Therefore, our results indicate that depending on type of cells these extracts may act as anti- or pro-oxidant factors. Most of cancer cells are characterized by lower levels of endogenous antioxidants such as glutathione peroxidase or catalase. Therefore, cancer cells may generate higher levels of ROS compared with properly functioning cells. Excessive ROS production may cause the damage to cellular structures, consequently leading to programmed cell death. There are also other studies that reveal ROS production as the inducer of cell cycle arrest at the G2/M phase [45]. Thus, it is possible that extract-induced cell cycle arrest may be mediated by stimulation of ROS production in cancer cells.

Surprisingly, purified forms of organic iodine were much less effective in inducing cell cycle arrest compared to control lettuce and iodine-fortified lettuce (Figure 2). One explanation could be that the effects of iodine-fortified lettuce are, at least partially, associated with polyphenols present in lettuce. Indeed, our previous study showed that biofortification of lettuce with the organic form of iodine has an influence on the concentration of polyphenolic compounds [12]. Our findings are consistent with the results of Kiferle [50] and Blasco [11], who also confirmed that using iodine increases content of phenolic compounds in lettuce and thereby improves the antioxidant response. Maglione et al. demonstrated that iodine treatment enhances accumulation of bioactive compounds such as polyphenols and carotenoids under condition of moderate salt levels [51]. Interestingly, they showed that the imposition of salinity in combination with iodine may enhance the activity of enzymes taking part in polyphenol synthesis and this effect may be potentiated by iodine [51]. It is thought that phenolic compounds can bind iodine by electrophilic substituting in the aromatic ring. Therefore, we hypothesized that more effective action of iodine fortified lettuce may results from the interactions between iodine and secondary metabolites occurring inside of the fortified plants. However, confirmation of this hypothesis requires further studies.

G2/M arrest observed upon treatments with the lettuce extracts suggests induction of cell death pathways. Indeed, the number of cells with inactive anti-apoptotic Bcl2 and with activated pro-apoptotic caspases was increased upon treatments with lettuce extracts with and without iodine fortification (Figure 3). These changes were associated with an increase in expression of pro-apoptotic genes such as *BAD, BAX, BID,* and *FAS*, and downregulation of anti-apoptotic *CCND1* and *CDK2* in AGS gastric cancer cells (Figure 4). The profile of expression changes in HT-29 colon cancer cells was distinct from AGS cells. In HT-29 cells, pro-apoptotic genes were mostly upregulated upon control lettuce with only *BAD* and *CCND1* affected upon the lettuce fortified with organic iodine vs. negative control (Figure 4). It could suggest that extracts from organic iodine-fortified lettuce lead to a switch from apoptosis towards cell cycle arrest and inhibition of proliferation. This suggestion is supported by increased expression of *CDKN2A*, a blocker of cell cycle progression [52], in HT-29 cells upon treatment with organic iodine-fortified lettuce vs. control lettuce (Figure 4). *CDKN2A* up-regulation upon extracts with organic iodine coincided with *MDM2* downregulation (Figure 5). It indicates that inhibition of HT-29 cell proliferation could be mediated through MDM2-p53 pathway, where *MDM2* acts as *p53* antagonist, although further studies are required to test this hypothesis.

The switch from pro-apoptotic to anti-proliferative effects of lettuce extracts in HT-29 cells is also supported by downregulation of key oncogenes, such as *KRAS*, *MDM2*, and *Myc* (Figure 5), which are implicated in oncogenic signaling pathways [53,54,55,56]. Apart from antagonizing *p53*, *MDM2*, for instance, is a downstream element of PI3K/AKT oncogenic pathway and part of MAPK and NF-κB signaling [54,55]. Another level of complexity is brought about by the involvement of *MDM2* in the epigenetic regulation of gene transcription. By negatively regulating *RB*, *MDM2* increases *DNMT3A* activity and DNA methylation-dependent silencing of tumor suppressor genes [29], as depicted in Figure 6C. In our present work, we observed that *MDM2* downregulation in HT-29 colon cancer cells (Figure 5B) is indeed associated with *RB1* upregulation upon 5-ISA-fortified lettuce extracts (Figure 4C). Further investigation confirmed DNA hypomethylation and upregulation of *SEMA3A* tumor suppressor gene (Figure 6A,B), promoter of which was shown to be bound by *DNMT3A* in cancer cells in our previous studies [30]. These findings suggest a possible role of MDM2/RB/DNMT3A-dependent pathway in the epigenetic effects of 5-ISA-fortified lettuce in HT-29 cells. According to a scheme in Figure 6C, *MDM2* downregulation upon 5-ISA-fortified lettuce results in *RB1* upregulation and *RB1*-dependent decrease in *DNMT3A* activity; thereby *SEMA3A* promoter is free of *DNMT3A* binding, promoter DNA methylation decreases and gene is transcribed. A mechanistic confirmation of this pathway needs to be addressed in future experiments.

Those epigenetic effects of 5-ISA-fortified lettuce may result from the action of polyphenols present in lettuce and specifically iodine-mediated enhancement of polyphenol content in lettuce [12]. Indeed, polyphenols from lettuce such as hydroxycinnamic acids, including chlorogenic acid, were shown to impact DNA methylation and DNA methylating enzymes in human cells [16,17,20]. Furthermore, the presence of salicylic acid, which is a defense and signaling plant hormone, may additionally contribute to the epigenetic impact of 5-ISA-fortified lettuce as salicylate’s derivatives, including olsalazine, were demonstrated to alter DNA methylation patterns in canine cancer cells [21]. In addition, treatment with salicylic acid selectively reduced DNA methylation of stilbene synthase genes activating them and thus increasing the production of resveratrol in plant cells [22], which may link salicylic acid to increased polyphenol content in 5-ISA-fortified lettuce as well. In terms of iodine presence in 5-ISA-fortified lettuce, there is little known about potential effects of iodine on DNA methylation in human cells although iodine affects thyroid function and play a role in thyroid cancer and other thyroid disease [23]. Nevertheless, iodine excess has been shown to affect DNA methylation of selected genes in blood of patients with autoimmune thyroiditis [24,25]. Hence, potential epigenetic effects of iodine cannot be ruled out.

Interestingly, 5-ISA-fortified lettuce led to a higher upregulation of *SEMA3A* compared with control lettuce, although the effect of the extracts on DNA hypomethylation was similar (Figure 6). It suggests that possibly additional epigenetic mechanisms are involved in the action of iodine-fortified lettuce, including histone marks or micro-RNA mechanisms. A wider spectrum of epigenetic effects of 5-ISA-fortified lettuce could be explained by the presence of salicylic acid. Indeed, a salicylic acid derivative, anacardic acid, acts as a histone deacetylase inhibitor in human cancer cells [26]. Another explanation could come from research on polyphenols where one of the major hydroxycinnamic acids in lettuce, chlorogenic acid, was identified as histone acetyltransferase inhibitor [27], and was shown to inhibit proliferation of colon cancer cells [18] and ameliorate other diseases [19] through micro-RNA-dependent mechanism.

The discrepancies in the effects produced by 5-ISA- or 3,5-diISA-fortified extracts on gene expression profiles may arise from the different molecular mechanisms by which these compounds lead to cell death. Identification of signaling pathways that regulate cell death is necessary to understand the difference in the effects between these compounds. In our previous studies, a significantly higher level of iodine was found in lettuce biofortified with 5-ISA and it was about 10-fold higher than in 3,5-diISA lettuce [13]. Therefore, diverse gene expression profiles may also be due to the different content of iodine in 5-ISA and 3,5-ISA-fortified lettuce extracts. Another reasons could include differences in the incorporation of the compounds into lettuce, type of tested cells or treatment durations.

The present study delivers novel evidence on distinct and cell-dependent anti-cancer effects of lettuce fortified with organic and inorganic forms of iodine. The findings suggest that fortified lettuce extracts exert mostly pro-apoptotic effects in AGS gastric cancer cells whereas anti-proliferative mode of action with the inhibition of oncogenes is predominant in HT-29 colon cancer cells. We show that lettuce fortified with an organic form of iodine, i.e., iodosalicylate 5-ISA, leads to epigenetic activation of DNA methylation-silenced *SEMA3A* tumor suppressor gene in HT-29 cells, which may be linked to MDM2/RB/DNMT3A pathway. Such effects could result from iodine-mediated increase in polyphenol content or salicylate action as both polyphenols and salicylic acid have been reported to produce epigenetic effects in human cells. Our findings provide foundation for further research on the mechanistic link between iodine, salicylate, and polyphenols in food extracts.

## 5. Patents

The method of enriching plants with iodine with: 5-iodosalicylic acid and 3,5-diiodosalicylic acid is patented by the Polish Patent Office-patent number P.410806 (20 November 2017).

## Figures and Tables

**Table 1:** Cytotoxicity of extracts from iodine-biofortified lettuce in human gastrointestinal cell line (AGS), colorectal adenocarcinoma cell line (HT-29) and normal colon epithelial cell line (CCD 841 CoN).

	Cytotoxicity [%]		
Treatment	AGS	HT-29	CCD 841 CoN
Control lettuce	1.84 ± 0.04	3.24 ± 0.01	3.27 ± 0.37
KIO_3_ lettuce	1.18 ± 0.07	2.95 ± 0.13	0.00 ± 0.14
5-ISA lettuce	0.11 ± 0.31	0.24 ± 0.06	5.69 ± 0.52
3,5-diISA lettuce	6.27 ± 0.08	1.00 ± 0.04	2.29 ± 0.76

## Data Availability

The Authors will share the data upon request.

## Supplementary Materials

The following supporting information can be downloaded at: https://www.mdpi.com/article/10.3390/nu14204287/s1, Figure S1: Cell cycle distribution from Muse^®^ Cell Cycle Assay; Figure S2: Representative plots from Muse^®^ Bcl-2 Activation Dual Detection Assay; Figure S3: Representative plots from Muse^®^ MultiCaspase Assay; Figure S4: Representative plots from Muse^®^ Annexin V & Dead Cell Assay; Table S1: Amplicon context sequences prepared by Bio-Rad used in gene expression analysis by qRT-PCR; Table S2: Primer sequences used in gene expression analysis by qRT-PCR; Table S3: Primer sequences used in DNA methylation analysis by pyrosequencing.
