# Supplementary material for: Molecular Effects of Iodine-Biofortified Lettuce in Human Gastrointestinal Cancer Cells
Source: Nutrients. 2022 Oct 14;14(20):4287. doi: 10.3390/nu14204287 (PMC9607317; doi:10.3390/nu14204287)
Supplement: Supplementary file 1 [file nutrients-14-04287-s001.zip › nutrients-1950076-supplementary.pdf]

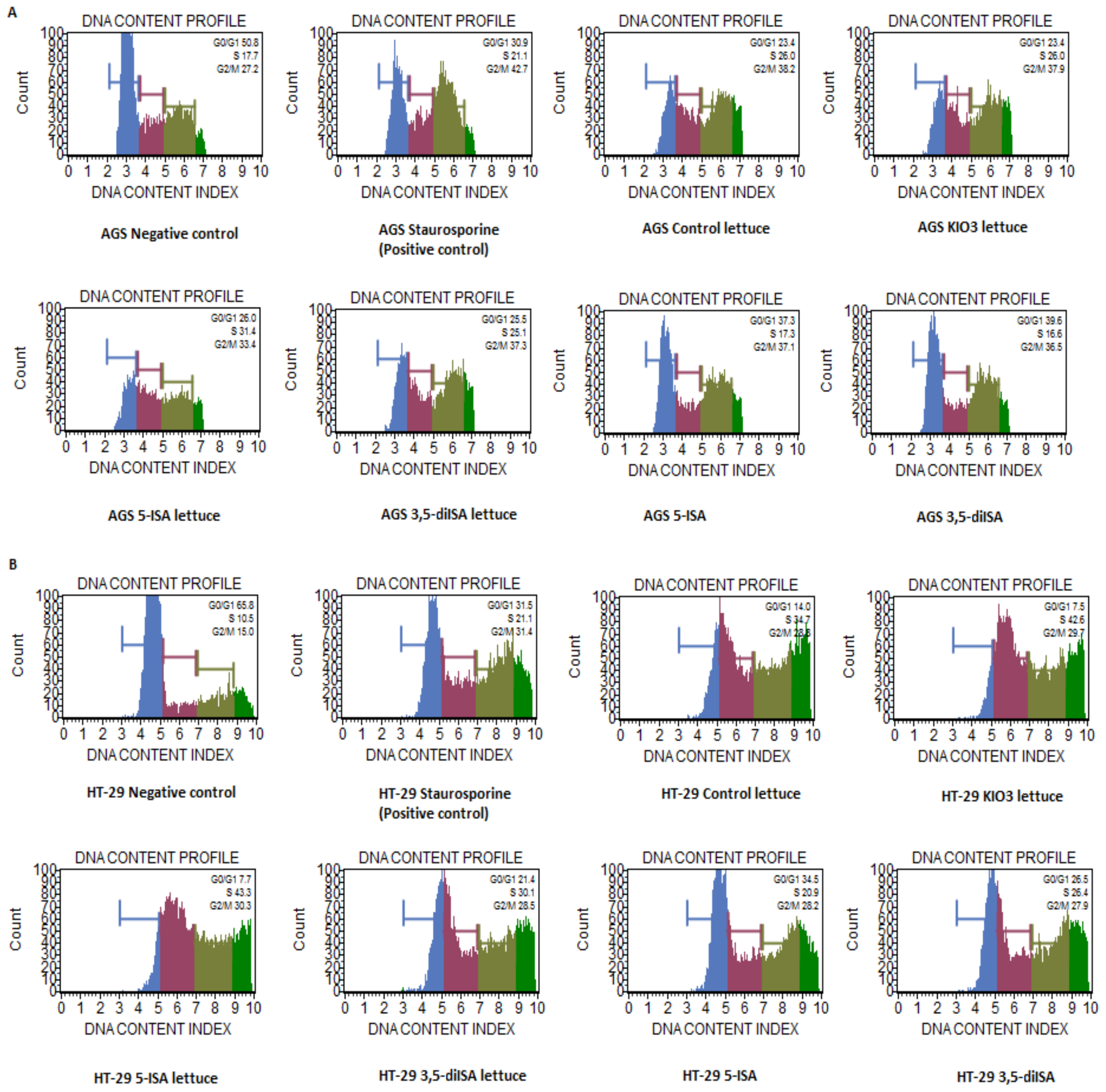

Supplementary Figure S1. Cell cycle distribution from Muse® Cell Cycle Assay. The effect of extracts from iodine-biofortified lettuce on cell cycle in human gastrointestinal cell line AGS **(A)** and colorectal adenocarcinoma cell line HT-29 **(B)**. The above plots show the histograms presenting DNA content index versus count for assessing the percentage of cells in each phase of cell cycle.

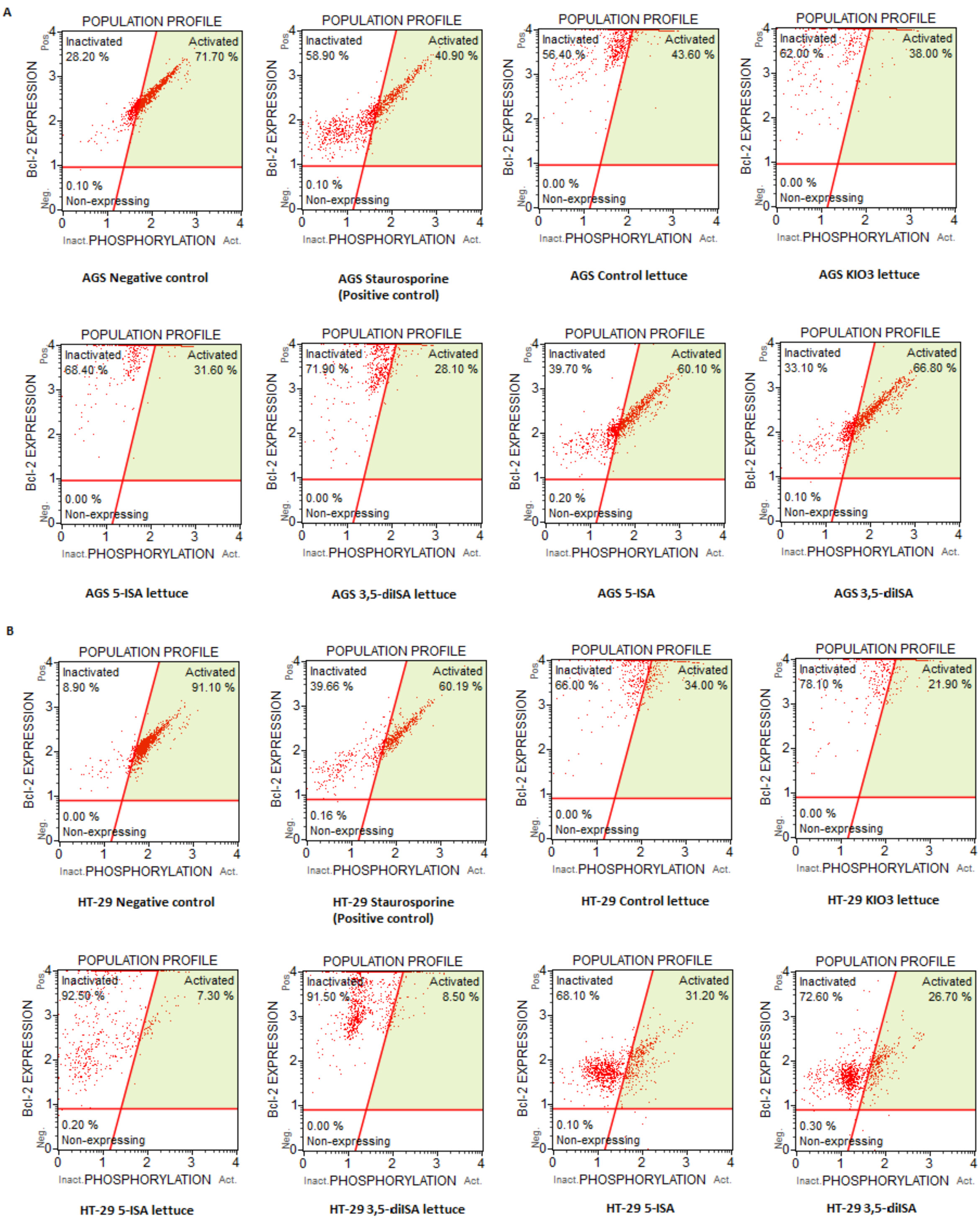

Supplementary Figure S2. Representative plots from Muse® Bcl-2 Activation Dual Detection Assay. The effect of extracts from iodine-biofortified lettuce on Bcl-2 expression in human gastrointestinal cell line AGS **(A)** and colorectal adenocarcinoma cell line HT-29 **(B)**. The above dot plots show cells which are inactivated, non-expressing and activated.

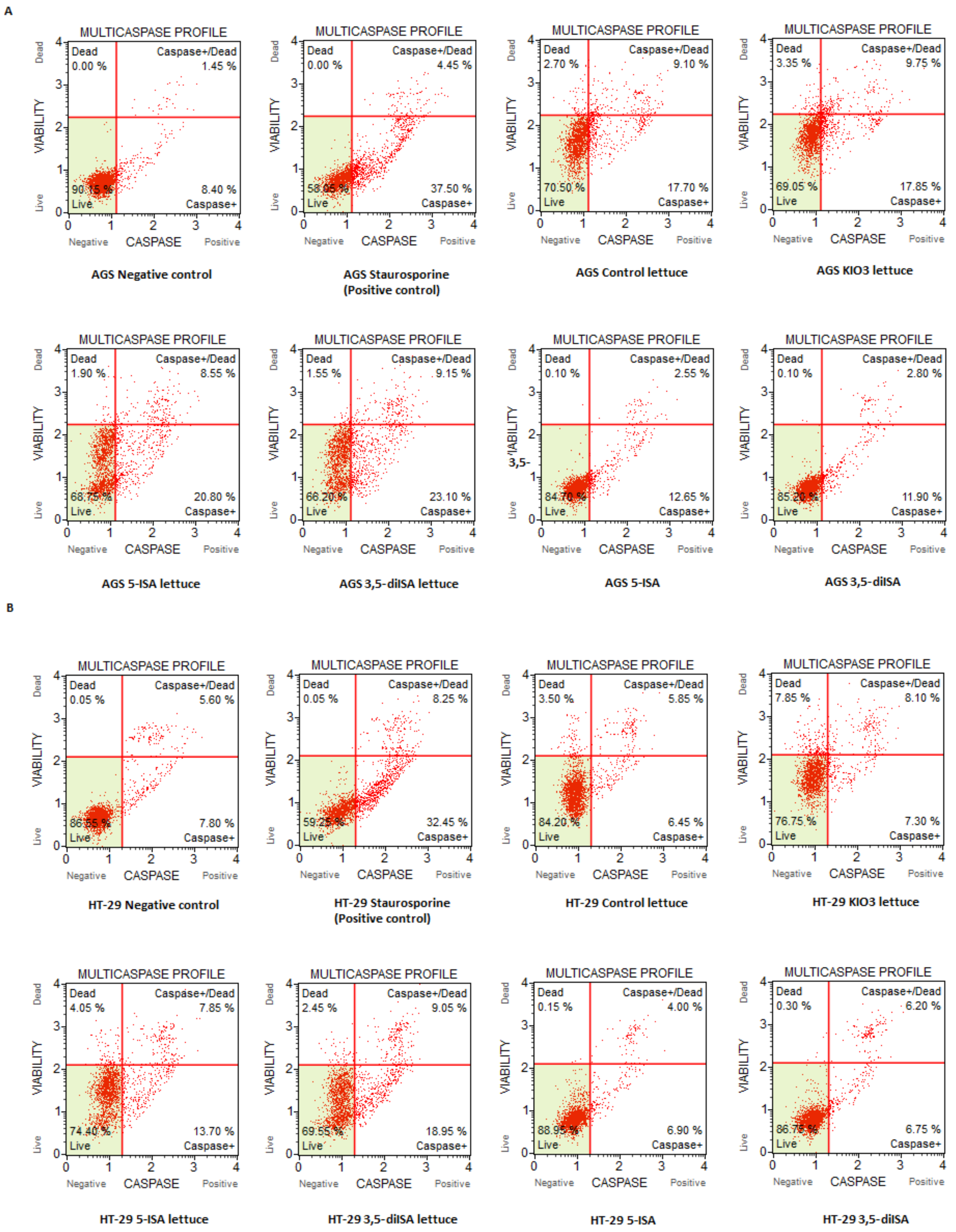

Supplementary Figure S3. Representative plots from Muse® MultiCaspase Assay. The effect of extracts from iodine-biofortified lettuce on activity of caspases in human gastrointestinal cell line AGS **(A)** and colorectal adenocarcinoma cell line HT-29 **(B)**. The above dot plots show four cell populations: Live, Caspase(+), Caspase(+)/Dead, and Dead cells.

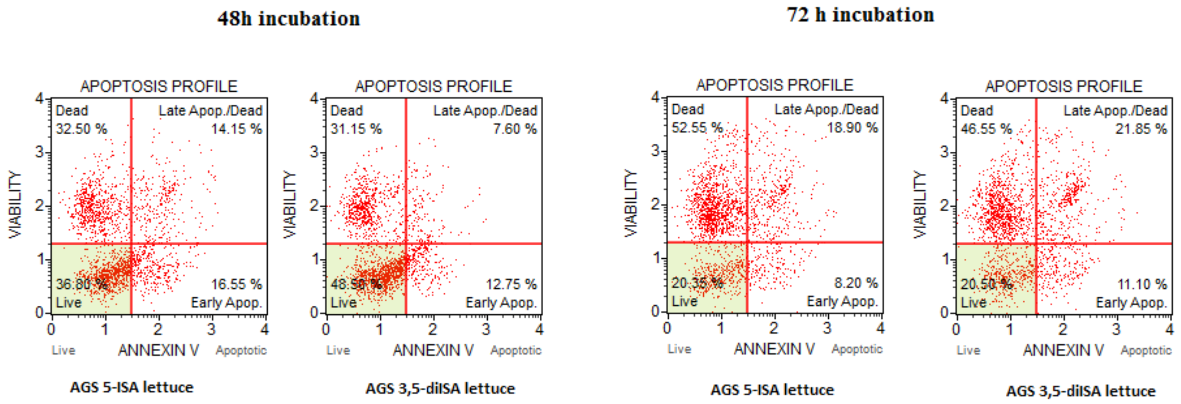

Supplementary Figure S4. Representative plots from Muse® Annexin V & Dead Cell Assay. The effect of extracts from iodine-biofortified lettuce on apoptosis in human gastrointestinal cell line AGS. The above dot plots show the histograms presenting four cell populations – live, early apoptotic, late apoptotic, and dead cells.

Supplementary Table S1. Amplicon context sequences prepared by Bio-Rad used in gene expression analysis by qRT-PCR.

Gene	Amplicon Context Sequence	Melting temperature [°C]	Amplicon length [bp]
RB1	CTGTCTGACTACTTTGCCTTCTTTTGTAGCATATAGGTGATGTTTG CTCTTGTTTTTATTAATTTATATGTATATTTTTTTAATTTAACATGA ACACCCTTAGAAAATGTGTCCTATCTATCTTCCAAATGCAAT	75	105
CDKN2A	ATTTGCTAGCAGTGTGACTCAAGAGAAGCCAGTAACCCCCCTGA GCTTCCCTAGTTCACAAAATGCTTGTCATGAAGTCGACAGCTTCC GGAGGCTGCGAGGCTCGC	81	77
BAD	ACAGACGCGGGCTTTATTAACATTTGGTAGTGAGCACGGCCCCC AGGGCATCGCGGGGGCTCGGGTCCCGGTGACGCAACGGTTAAA CCTGGCTCGCGACTTAGCGCAGGCGCCTGGGGGAAAG	88.5	94
BAX	GCACCAAGGTGCCGGAACTGATCAGAACCATCATGGGCTGGAC ATTGGACTTCCTCCGGGAGCGGCTGTTGGGCTGGATCCAAGACC AGGGTGGTTGGGTG	83.5	71
BID	CATTTCTGGCTAAGCTCCTCACGTAGGTGCGTAGGTTCTGGTTAA TAAAATTCACTGTTGTGTGAAAGACATCACGGAGCAAGGACGG CGTGTGACTGG	79.5	69
FAS	AATGAAGCCAAAATAGATGAGATCAAGAATGACAATGTCCAAG ACACAGCAGAACAGAAAGTTCAACTGCTTCGTAATTGGCATCA ACTTCATGGAAAGAAAGAAGCGTATGACACATTGATTAAAGA	79.5	98
CCND1	TGAAGGCGGAGGAGACCTGCGCGCCCTCGGTGTCCTACTTCAAA TGTGTGCAGAAGGAGGTCCTGCCGTCCATGCGGAAGATCGTCGC CACCTGGATGCTGGAGGTCTGCGAGGAACAGAAGTGCGAGGAG GAGGTCTTCCCGCTGGCCATGAACTACCTGGACCGCTTCCTG	87.5	143
CDK2	CAGATGAGGTGGTGTGGCCAGGAGTTACTTCTATGCCTGATTAC AAGCCAAGTTTCCCCAAGTGGGCCCGGCAAGATTTTAGTAAAGT TGTACCTCCCCTGGATGAAGATGGACGGAGCTTGTTATCGCAAA TGCTGC	82.5	107
AKT1	ATGGTGATCATCTGGGCCGTGAACTCCTCATCAAAATACCTGGT GTCAGTCTCCGACGTGACCTGGGGCTTGAAGGGTGGGCTGAGCT TCTTCTCGTACACGTGCTGCCACACGATACCGGCAAAGAAGCGA TGCTGCATGATCTCCTTGGCGTCCTCGGAGCCCCCGCCAAG	86.5	143

HRAS	TTGGTGTTGTTGATGGCAAACACACACAGGAAGCCCTCCCCGGT GCGCATGTACTGGTCCCGCATGGCGCTGTACTCCTCCTGGCCGG CGGTATCCAGGATGTCCAACAGGCACGTCTCCCCATCAATGA	87.5	100
KRAS	CAAAAGCTTCATTAATTTGTTTCACACCAACATTCACAATTGGTA AGAAAAATAAGAAGTAATCAACTGCATGCACCAAAAGCCCCAA GACAGAAATCTTAGGTATTCAGTTTCTTTTTCACAGGCATTGCTA GTTCAAAAACCAAAACT	86.5	120
NRAS	GTTTTTTCATTCGGTACTGGCGTATTTCTCTTACCAGTGTGTAAAA AGCATCTTCAACACCCTGTCTGGTCTTGGCTGAGGTTTCAATGAA TGGAATCCCGTAACTCTTGGCCAGTTCGTGGGCTTGTTTTGTATC AACTGT	81.5	112
MDM2	AACCCAAGACAAAGAAGAGAGTGTGGAATCTAGTTTGCCCCTT AATGCCATTGAACCTTGTGTGATTTGTCAAGGTCGACCTAAAAA TGGTTGCATTGTCCATGGCAAAACAGGACATCTTAT	80.5	93
MYC	CCGCCCACCACCAGCAGCGACTCTGAGGAGGAACAAGAAGATG AGGAAGAAATCGATGTTGTTTCTGTGGAAAAGAGGCAGGCTCCT GGCAAAAGGTCAGAGTCTGGATCACCTTCTGCTGGAGGCCACA GCA	82.5	103

Supplementary Table S2. Primer sequences used in gene expression analysis by qRT-PCR.

**Gene**	**Primer sequences**	**Annealing temperature [°C]**	**Amplicon length [bp]**
SEMA3A	FW 5'-TAGGCTGTATGTTGGAGCAAAG-3'<br>RV 5'-AGCCCACTTGCATTCATCTC-3'	59	118

Supplementary Table S3. Primer sequences used in DNA methylation analysis by pyrosequencing.

**Gene**	**Primer sequences**	**Annealing temperature [°C]**	**Amplicon length [bp]**
SEMA3A	FW 5'-GGGGGATTTTTAAAAGGATATTTAGA-3'<br>RVBio 5'-AAAACCACAACCAACTACTTATTT-3'<br>Seq 5'-GGTTATTAAATTTTTTGTGGG -3'	50	321
